# Replacing sedentary time with physical activity: effects on health-related quality of life in older Japanese adults

**DOI:** 10.1186/s12955-018-1067-8

**Published:** 2018-12-27

**Authors:** Akitomo Yasunaga, Ai Shibata, Kaori Ishii, Shigeru Inoue, Takemi Sugiyama, Neville Owen, Koichiro Oka

**Affiliations:** 1 Faculty of Liberal Arts and Sciences, Bunka Gakuen University, 3-22-1 Yoyogi, Shibuya-ku, Tokyo Japan; 2 Faculty Health and Sport Sciences, University of Tsukuba, Tsukuba, Ibaraki Japan; 3 Faculty of Sport Sciences, Waseda University, Tokorozawa, Saitama Japan; 4 Department of Preventive Medicine and Public Health, Tokyo Medical University, Shinjuku-ku, Tokyo Japan; 5 Mary MacKillop Institute for Health Research, Australian Catholic University, Melbourne, VIC Australia; 6 Centre for Urban Transitions, Swinburne University of Technology, Hawthorn, VIC Australia; 7 Behavioural Epidemiology Laboratory, Baker Heart and Diabetes Institute, Melbourne, VIC Australia

**Keywords:** Accelerometer, Physical health, Lifestyle activity, Mental health, Sitting

## Abstract

**Background:**

The isotemporal substitution (IS) approach can be used to assess the effect of replacing one activity with the equal duration of another activity on relevant outcomes. This study examined the associations of objectively assessed sedentary behavior (SB) and physical activity (PA) with health-related quality of life (HRQOL) in older Japanese adults, using the IS approach.

**Methods:**

Participants were 287 older Japanese adults (aged 65–84 years) who wore accelerometers for at least 7 days. We calculated the average daily time spent in SB (≤1.5 METs); light-intensity PA (LPA: > 1.5 to < 3.0 METs); and moderate- to vigorous-intensity PA (MVPA: ≥3.0 METs) per day. HRQOL was assessed using the Medical Outcomes Survey Short Form-8 questionnaire.

**Results:**

The IS models showed replacing SB or LPA with MVPA to be significantly associated with better physical component summary scores. Replacing SB with MVPA was marginally associated with better mental component summary scores.

**Conclusion:**

These findings indicate that replacing SB with the same amount of MVPA may contribute to better physical HRQOL in older adults.

## Background

Health-related quality of life (HRQOL) defined as “a measure of person’s physical, psychological, and social aspects of health, reflecting personal beliefs, preferences, experiences, and perceptions” [1], is known to be associated with mortality, chronic diseases, and risk for disability among older adults [2–4]. In the context of declining physical function and high prevalence of mental health problems among older adults [5, 6], maintaining better quality of life in later life is an important goal for countries experiencing rapid population aging [7].

Engaging in regular moderate- to vigorous-intensity physical activity (MVPA) is known to be positively related to HRQOL [8]. One cross-sectional study found associations of self-reported physical activity (PA) and pedometer steps with HRQOL among a population-based sample of adults over 55 years [9]. Recent studies have shown that light-intensity PA (LPA; e.g., housework, gardening, and casual walking) and sedentary behavior (SB; e.g., television viewing, computer use, workplace sitting, and sitting in automobile) are also related to health of older adults. A systematic review reported that light-intensity PA (1.5–3.0 metabolic equivalent tasks [METs]) can improve physical and cognitive health of older adults [10]. SB has been shown to be detrimentally associated with various health outcomes, after controlling for the role of participation in MVPA [11–13].

However, there is a methodological concern in previous studies examining the relationships between PA of different intensity levels and HRQOL. SB, LPA, and MVPA are all parts of daily life and can be inter-related: increasing one behavior will result in a reduction of other behaviors, each of which may have impact on HRQOL. Therefore, such complementary relationships between behaviors need to be considered when research examines how a particular activity is associated with outcomes such as HRQOL. However, the potential impact of replacing one behavior with another on older adults’ HRQOL is less well known.

We used the isotemporal substitution (IS) approach, a statistical method to evaluate the impact of replacing one activity with an equal duration of another activity, to examine the relationships of objectively assessed SB, LPA, and MVPA with HRQOL in a sample of Japanese older adults.

## Methods

### Participants and procedures

Detailed methods of this study have been described elsewhere [14]. Briefly, data were collected between October and December 2013 from adults aged 65–84 years living in Matsudo (population in 2013: approx. 480,000), Chiba Prefecture, Japan. A total of 3000 residents were randomly selected from the registry of residential addresses (stratified by gender and age groups), and sent a postal survey. Of 1250 who responded to the survey, those who had no mobility limitations were asked to take part in a sub-study using accelerometers. The sub-study participants (*n* = 349) received a book voucher worth approximately US$4 for participation.

### Measurements

#### Physical activity and sedentary behavior

Participants’ PA was assessed using Active style Pro HJA-350IT (Omron Healthcare, Kyoto, Japan). The detailed algorism and validity of the accelerometer have been described elsewhere [15]. Previous studies reported the accuracy of the accelerometer in assessing the intensity of PA for healthy older adults [16]. To be eligible, participants needed to wear the accelerometer device for ≥4 days (including 1 weekend day), with at least 10 h/day of wear time each day [17]. Non-wear time was identified using the criteria employed in previous research [17]. Using device’s internal thresholds, accelerometer output was classified into three levels: SB (≤1.5 METs); LPA (> 1.5 to < 3.0 METs); and MVPA (≥3.0 METs).

#### Health-related quality of life

HRQOL was assessed using the validated Japanese version of the Medical Outcomes Survey Short Form-8 questionnaire (SF-8) [18]. We aggregated the physical component summary (PCS) and mental component summary (MCS) following the scoring manual. Higher scores indicated better HRQOL.

#### Covariates

Participants reported age (years), gender, marital status, highest educational attainment, and disease history (the number of past illnesses, complications and comorbidity). Body mass index was calculated based on measured height and weight. These were adjusted for in this study as they have been shown related to PA, SB, and HRQOL in previous studies [19–21].

### Statistical analyses

We conducted three multiple linear regression models, single-activity model, partition model, and IS model, to assess the cross-sectional associations of SB, LPA, and MVPA with the two summary scores of the SF-8. We used 10 min/day as a unit for activity, as this is the minimum amount of time through which activities should be accrued to meet current PA guidelines [22].

The single-activity model assessed each activity component separately, only adjusting for total wear time and confounders. The model (in the case of SB) is expressed as follows:

$$ \mathrm{Outcome}\ \mathrm{variable}=\left({\mathrm{b}}_1\right)\ \mathrm{SB}+\left({\mathrm{b}}_4\right)\ \mathrm{total}\ \mathrm{wear}\ \mathrm{time}+\left({\mathrm{b}}_5\right)\ \mathrm{covariates}. $$

The coefficient b_1_ represents the effect of increasing SB, while holding the total time constant, without specifying a behavior to be replaced. The partition model examined all behaviors simultaneously, without adjusting for total wear time. It is expressed as follows:

$$ \mathrm{Outcome}\ \mathrm{variable}=\left({\mathrm{b}}_1\right)\ \mathrm{SB}+\left({\mathrm{b}}_2\right)\ \mathrm{LPA}+\left({\mathrm{b}}_3\right)\ \mathrm{MVPA}+\left({\mathrm{b}}_5\right)\ \mathrm{covariates}. $$

In this model, the coefficient for one type of activity represents the effect of increasing this type of activity while holding the other activities constant. Since total wear time is not included in the model, it represents the effects of simply adding the activity without substitution. The IS model specifies a “target” behavior that is to be replaced by a behavior of interest, while holding the total time constant. This can be accomplished by omitting the target behavior from the model and entering total wear time in the model. The IS model (SB as the target behavior) is expressed as follows:

$$ \mathrm{Outcome}\ \mathrm{variable}=\left({\mathrm{b}}_2\right)\ \mathrm{LPA}+\left({\mathrm{b}}_3\right)\ \mathrm{MVPA}+\left({\mathrm{b}}_4\right)\ \mathrm{total}\ \mathrm{wear}\ \mathrm{time}+\left({\mathrm{b}}_5\right)\ \mathrm{covariates}. $$

The coefficient b_2_, for instance, can be interpreted as the effect of replacing LPA with the same duration of SB, since MVPA and total wear time are held constant. Statistical significance was set at the 0.05 level. We conducted all analyses using IBM SPSS Statistics 20.0 (IBM Japan Corp., Tokyo).

## Results

### Characteristics of study participants

After excluding participants without valid PA accelerometer data and covariates, the final sample consisted of 287 participants. The characteristics of the participants are shown in Table 1. The mean number of valid days of accelerometer wear was 7.2 (SD, 0.9). The mean proportion of each behavior to the total accelerometer wear time was 58% for SB, 36% for LPA, and 6% for MVPA. Correlation coefficients between the three activity variables were − 0.67 between SB and LPA, − 0.35 between SB and MVPA, and 0.23 between LPA and MVPA.

**Table 1:** Participant characteristics (*n* = 287)

	M (SD) or n %	
Gender (men)	179	62.4%
Age (years)	74.4	(5.2)
Marital status		
Married	238	82.9%
Widowed, divorced, separated, or never married	49	17.1%
Highest educational attainment		
University, junior college, vocational school, or higher-level degree	111	38.7%
High school or lower	176	61.3%
The number of past illnesses, complications and comorbidity	1.6	(0.9)
Body mass index (kg/m^2^)	23.4	(3.2)
Activity		
SB (min/day)	524.2	(113.0)
LPA (min/day)	328.5	(101.4)
MVPA (min/day)	50.0	(32.5)
Total wear time (min/day)	902.2	(87.0)
Medical Outcomes Survey Short Form-8		
Physical component summary (PCS)	47.9	(7.0)
Mental component summary (MCS)	51.8	(5.8)

*Note*. *SB* sedentary behavior, *LPA* light-intensity physical activity, *MVPA* moderate- to vigorous-intensity physical activity

### Associations of activity variables with HRQOL

The results of regression analyses (the single-activity, partition, and IS models) are shown in Table 2. In the single-activity model, SB was significantly and inversely associated with the PCS score. No significant association was observed for LPA. MVPA was significantly associated with better PCS score. No significant association was found for the MCS score in the single-activity models, but its association with MVPA approached significance (*p* = 0.09). In the partition model, MVPA was the only activity that was significantly associated with the PCS and marginally with MCS (*p* = 0.07) scores. In the IS model where SB was the target behavior, replacing it with MVPA was significantly and favorably associated with the PCS score. Similarly, replacing LPA with MVPA was associated with higher PCS scores. No significant association was observed for the MCS score in the IS models. However, replacing SB with MVPA had a marginally significant association with better MCS scores (*p* = 0.09). Other behavioral substitutions (e.g., SB with LPA and vice versa) were not associated with the PCS or MCS scores.

**Table 2:** Single-activity, partition, and IS models examining the associations of SB, LPA, and MVPA with HRQOL scores

	Model	Target behavior (to be replaced)	b (95%CI)		
			SB	LPA	MVPA
PCS	Single-activity	–	−0.09 (− 0.18, − 0.01)*	0.06 (− 0.04, 0.15)	0.45 (0.19, 0.72)**
	Partition	–	0.02 (− 0.08, 0.11)	0.05 (− 0.07, 0.16)	0.45 (0.18, 0.73)**
	IS	SB	Dropped	0.03 (−0.07, 0.13)	0.44 (0.17, 0.71)**
		LPA	−0.03 (− 0.13, 0.07)	Dropped	0.41 (0.11, 0.71)**
		MVPA	−0.44 (− 0.70, − 0.17)**	−0.41 (− 0.71, − 0.11)**	Dropped
MCS	Single-activity	–	− 0.02 (− 0.10, 0.05)	0.00 (− 0.08, 0.08)	0.20 (− 0.03, 0.42)†
	Partition	–	0.02 (− 0.06, 0.11)	0.01 (− 0.09, 0.11)	0.23 (− 0.02, 0.47)†
	IS	SB	Dropped	− 0.01 (− 0.10, 0.07)	0.20 (− 0.03, 0.43)†
		LPA	0.01 (− 0.07, 0.10)	Dropped	0.21 (− 0.05, 0.47)
		MVPA	−0.20 (− 0.43, 0.03) †	−0.21 (− 0.47, 0.05)	Dropped

† *p* < 0.1, * *p* < 0.05, ** *p* < 0.01

Note. *PCS* Physical component summary, *MCS* Mental component summary, *SB* sedentary behavior, *LPA* light-intensity physical activity, *MVPA* moderate- to vigorous-intensity physical activity

Regression coefficients correspond to a 10-min/day increment of each activity. Bold font denotes significant association

All models adjusted for age (years), gender, marital status (married/widowed, divorced, separated, or never married), highest educational attainment (up to and including high school/university, junior college, vocational school, or higher degree), the number of past illnesses, complications and comorbidity, and BMI (kg/m^2^). The single-activity and IS models also adjusted for total accelerometer wear time

## Discussion

This study examined how SB, LPA, and MVPA are related to older adults’ HRQOL using the IS approach. In discussing findings obtained from the IS models, we use terms such as “impact” and “effect”, since each IS model is to understand what may happen if one behavior is replaced with another. However, they do not imply any causal relationships.

Consistent with previous studies [23, 24], this study found that engaging in MVPA was associated with higher PCS scores (better QOL related to physical health). Significant associations were found consistently in the single-activity, partition, and IS models. In particular, IS models showed that MVPA replacing any behavior was associated positively with the PCS score. In contrast, the single-activity and partition models produced different results for SB. It was associated negatively with the PCS score in the single-activity model (total time held constant), while no significant association with the PCS score was found in the partition model (other activities held constant, no substitution). These findings suggest that the impact of SB may be partly due to the displacement of PA by SB. However, this is not consistent with previous studies showing detrimental associations of SB with health outcomes independent of MVPA [13, 17].

Another finding that is not consistent with existing research is the role of LPA. LPA was mostly unrelated to health outcomes in our study, but previous research has shown positive health impacts of LPA on older adults [25, 26]. Reasons for these unexpected findings are not known. However, LPA assessed in this study may have included household chores (e.g., cooking, washing dishes, ironing, gardening, and housecleaning). Since these chores are often “required” activity [27] in contrast to MVPA, which more-typically takes place in leisure settings [28], a large amount of time spent in LPA potentially could increase stress and strain in some older adults, rather than being beneficial to their HRQOL. It is important to understand whether and to what extent LPA is beneficial to older adults’ physical and mental health. Further research exploring the content/setting of behaviors may provide insights into this issue.

We did not find significant associations of the MCS score (QOL related to mental health) with any of the behaviors examined, except for marginal associations with MVPA. A meta-analysis examining the link between PA and psychological well-being in older adults [29] reported that moderate PA benefits older adults’ well-being. It is unknown why this study did not find associations of MVPA with better QOL related to mental health. The volume of MVPA was relatively large in this study. Moderate-intensity activities captured by the device may have contained some chores that may not be conducive to better mental health.

Limitations of this study include that it cannot infer causal impact of behaviors on HRQOL: it is possible that those with better health status were more physically active. Our findings may not be generalizable to the Japanese population, as data were collected from a relatively small sample. Further research using a longitudinal design with a larger sample size is needed to better understand how different levels of activity contribute to older adults’ HRQOL.

## Conclusions

In summary, our findings emphasize the importance of MVPA in older adults’ QOL related to physical health. We found that LPA was unlikely to contribute to better health-related QOL in this sample of Japanese older adults. Future research may need to investigate not only the intensity of physical activity but also its type and setting, in order to better understand how different aspects of PA and SB may be related to older adults’ well-being.

## Abbreviations

HRQOL: Health-related quality of life
IS: Isotemporal substitution
LPA: Light-intensity physical activity
MCS: Mental component summary
METs: Metabolic equivalent tasks
MVPA: Moderate- to vigorous-intensity physical activity
PA: Physical activity
PCS: Physical component summary
SB: Sedentary behavior
